# Inflammatory Subphenotypes for Fluid Management in Acute Respiratory Distress Syndrome: A Subgroup-Level Signal, not an Individual Prescription

**DOI:** 10.1097/CCE.0000000000001484

**Published:** 2026-09-07

**Authors:** Joris Pensier, Emmanuel Lorne

**Affiliations:** 1 Department of Anesthesia, Critical Care and Pain Medicine, Center for Anesthesia Research Excellence, Beth Israel Deaconess Medical Center, Harvard Medical School, Boston, MA.; 2 Anesthesiology and Intensive Care Department, Saint Eloi Teaching Hospital, Centre Hospitalier Universitaire Montpellier, University of Montpellier, INSERM U1046, CNRS UMR 9214, PhyMedExp, Montpellier, France.; 3 Anesthesia and Critical Care Department B, Saint Eloi Teaching Hospital, Centre Hospitalier Universitaire Montpellier, University of Montpellier, INSERM U1046, CNRS UMR 9214, PhyMedExp, Montpellier, France.; 4 Department of Anaesthesiology and Critical Care Medicine, Clinique du Millénaire, Montpellier, France.

**Keywords:** acute respiratory distress syndrome, echocardiography, phenotyping, precision medicine, right ventricular dysfunction

A decade ago, Famous et al (1) reported that the two inflammatory subphenotypes of acute respiratory distress syndrome (ARDS) responded in opposite directions to randomized fluid management. In a secondary analysis of the Fluid and Catheter Treatment Trial, a liberal fluid strategy was associated with lower 90-day mortality in the hyperinflammatory subphenotype (40% vs. 50% with conservative fluids) and conversely with higher mortality in the hypoinflammatory subphenotype (26% vs. 18% with conservative fluids). This subphenotype-treatment interaction was among the first signals that an inflammatory label might do more than prognosticate: it might tell clinicians how to treat. The subphenotypes differed in systemic inflammation, endothelial injury, and hemodynamic and renal function, so a differential fluid response seemed physiologically plausible. As fluid therapy is one of the most consequential and least standardized decisions in the ICU, the perspective of phenotype-guided fluid therapy was especially attractive. Yet a differential average treatment effect across subgroups and a rule that can be applied to the patient in front of us are not the same thing. The former is a property of a population, whereas the latter is a claim about an individual. In this issue of *Critical Care Explorations*, Chotalia et al (2) give us the material to seek whether this signal can guide the individual patient or only describe the group.

The authors applied an independently validated, open-access clinical classifier for inflammatory subphenotypes (https://bostonmontpelliercare.shinyapps.io/AIClarity/; 3, 4) to 691 intubated patients with ARDS who had undergone standardized transthoracic echocardiography. They then compared cardiovascular parameters between the hyper- and hypoinflammatory groups; the results give inflammation a hemodynamic face. Focusing on the left ventricle and systemic circulation, the hyperinflammatory subphenotype was distinctly hyperdynamic (2): compared with the hypoinflammatory subphenotype, it showed higher left ventricular (LV) ejection fraction (70% vs. 65%; *p* < 0.0001) and cardiac index (4.2 L/min/m^2^ vs. 3.2; *p* < 0.0001), lower systemic vascular resistance index (1038 dynes·s/cm^5^ vs. 1704; *p* < 0.0001), and a smaller, more collapsible inferior vena cava. At the subphenotype level, 47% of the hyperinflammatory patients were classified in the hyperdynamic cardiovascular subphenotype, vs. 11% of the hypoinflammatory patients. This portrait appears at first glance to explain Famous et al’s results (1). A vasoplegic, high-output circulation with a collapsing vena cava is, at the group level, the picture of relative underfilling, a physiology one would expect to tolerate or require volume. The mechanistic loop seems to close: the hyperinflammatory patient has a hyperdynamic circulation, therefore benefits from a liberal strategy. Conversely, a restrictive strategy in hypoinflammatory patients allows for lower fluid balance and faster weaning from the ventilator. But the authors go further, using latent class analysis of echocardiographic and hemodynamic variables to recover four previously described cardiovascular subphenotypes (5): “normal function,” “hyperdynamic cardiovascular function,” “right ventricular (RV) dysfunction,” and “RV failure.”

The hyperdynamic picture captures the left ventricle and the systemic vasculature, but does not extend to the right ventricle. At the bedside, especially when ARDS patients are exposed to high positive end-expiratory pressure (PEEP), it is often RV tolerance that sets the ceiling on how much fluid a patient can safely receive (6). In the present cohort, RV size and function did not differ between the hyper- and hypoinflammatory subphenotypes, and there was no interaction between the RV-related cardiovascular and inflammatory subphenotypes (2). The authors therefore demonstrate that systemic inflammation and RV dysfunction are orthogonal: knowing one tells you nothing about the other. In their earlier echocardiographic work, RV failure was the dominant cardiovascular determinant of mortality in ARDS, carrying a seven-fold increase in the odds of death (5). Together, these results break the step from group to individual. Inside the hyperinflammatory group sits a subset with occult RV failure for whom liberal fluids may be the least safe option. The inflammatory label, without physiologic monitoring, cannot identify them: the population average and the individual’s needs diverge. The assumption that a more hyperdynamic profile at the subphenotype level is enough to adopt a liberal fluid strategy in individuals becomes fragile, especially in the ventilated patient. In ARDS, etiology, loading conditions, intrathoracic pressure, and RV afterload confound any simple association between the level of systemic inflammation and the need for fluids.

A similar lesson emerged when inflammatory subphenotypes were examined through the lens of partitioned respiratory mechanics, in ARDS patients monitored with esophageal manometry (7). A previous secondary analysis of the ALVEOLI (Assessment of Low Tidal Volume and Elevated End-Expiratory Volume to Obviate Lung Injury) trial (8) had found that higher PEEP yielded better outcomes in hyperinflammatory patients, while lower PEEP appeared safer in hypoinflammatory patients. However, physiologically, the optimal PEEP differed only by 1 cm H_2_O, a statistically significant difference overshadowed by a clinically large interindividual variability (7). Inflammatory subphenotypes capture changes in chest wall mechanics plausibly reflecting endothelial impairment and fluid balance; yet these changes are insufficient in magnitude and too variable between patients to guide a specific PEEP strategy. The present study extends this principle from ventilation to fluid therapy: hyperinflammation may point toward a vasoplegic, hyperdynamic circulation at the group level (2). But fluid tolerance in an individual patient depends on the broader cardiovascular dimension: preload reserve, venous congestion, pulmonary vascular load, and especially RV tolerance. Thus, respiratory and cardiovascular physiology do not compete with inflammatory phenotyping; they refine it (9). The phenotype provides a biological prior: phenotyping can help understand why a treatment signal emerges at the subgroup level and can identify patients in whom a given therapeutic axis deserves attention. But the final therapeutic strategy must be individualized by the patient’s measured physiology.

Several strengths deserve emphasis. First, the study brings advanced, standardized echocardiographic phenotyping to a large cohort of intubated ARDS patients, with examinations performed by highly trained operators, and a clinical classifier serving as a bridge to connect the cardiovascular subphenotypes to inflammatory subphenotypes. This allowed the study to complement, extend, and contextualize the landmark findings of Famous et al (1). Cardiovascular phenotyping was not reduced to isolated echocardiographic variables, but embedded within a previously described latent-class framework, allowing the authors to compare coherent, multidimensional physiologic profiles. The authors also strengthen the analysis by reporting *E*-values, which help quantify the robustness of the observed associations. For the association between hyperinflammation and the hyperdynamic cardiovascular subphenotype, the *E*-value of 4.9 supports a strong subgroup-level relationship: an unmeasured factor would need to be associated with both the inflammatory and cardiovascular subphenotypes by a risk ratio of at least 4.9 to fully explain away the association. The limitations are equally important for interpretation. The study is single-center and retrospective, and inclusion required the availability of transthoracic echocardiography, which may have selected patients in whom cardiovascular assessment was already clinically relevant. Phenotyping was also performed at a single time point, whereas inflammatory subphenotypes are dynamic, with transitions occurring over the first days of ARDS (3, 10). How inflammatory and cardiovascular subphenotypes co-evolve over time therefore remains unknown, although this longitudinal interaction may be clinically consequential (11). Most importantly, the echocardiographic protocol did not include RV-to-pulmonary-artery coupling, or direct measures of fluid responsiveness and venous congestion. The lack of pulmonary artery systolic pressure (PASP), pulmonary vascular resistance (PVR), coupling indices (tricuspid annular plane systolic excursion [TAPSE]/PASP, effective arterial elastance/end-systolic elastance), and detailed ventilatory pressures prevents distinguishing afterload-driven ventricular dysfunction (pulmonary vascular vs. mechanical) from intrinsic cardiac dysfunction or volume status. Without RV or LV strain or septal shape assessment, subclinical RV dysfunction or prognostic paradoxical motion goes undetected. The hemodynamic picture remains purely descriptive, unable to mechanistically guide therapy (vasopressor, inotrope, ventilator settings). Thus, the physiologic variables recorded here characterize cardiovascular profiles, but cannot directly translate into bedside management.

More broadly, not all heterogeneity-of-treatment-effect findings across inflammatory subphenotypes are equally actionable. A signal may transfer better to the individual when the treatment acts on the same causal axis that defines the subphenotype (12). When the intervention targets immunomodulation, the logic is relatively direct: corticosteroids appeared beneficial in hyperinflammatory ARDS and harmful in hypoinflammatory ARDS (3), and simvastatin showed a similar phenotype-dependent effect (13). These findings are biologically consistent with the immune and metabolic differences described between subphenotypes (14). By contrast, when the intervention acts on a physiologic axis distinct from inflammation, such as fluid management or PEEP titration, the inflammatory label may be only a partial proxy for the true effect modifier. Fluid management is the paradigm case, and this study shows why.

In their study, Chotalia et al (2) dissect the cardiovascular dimensions of inflammatory subphenotypes and show that hyperinflammation aligns with a hyperdynamic systemic circulation, but not with RV-related cardiovascular profiles. The clinical consequence is that titrating fluids by inflammatory subphenotype alone would be an unsupported jump from subgroup signal to individual prescription. As a corrective, one should place inflammatory enrichment alongside the axis it cannot see: direct physiologic assessment. The path toward true precision medicine in ARDS likely lies in multidimensional, time-updated phenotyping that combines biological, physiologic, and morphological axes. The key question then is to match each treatment to the axis it truly modifies: immune interventions may be guided by inflammatory biology interpreted in clinical context, whereas ventilatory and fluid strategies must be grounded in measured, individual physiology.
